# Réaction à Mohamed Said Nakhli et al. à propos de l'article: “Quand le bloc axillaire reste la seule alternative chez un enfant de 5 ans”. The Pan African Medical Journal. 2015;21:36

**DOI:** 10.11604/pamj.2017.28.159.7174

**Published:** 2017-10-19

**Authors:** Anouar Jarraya

**Affiliations:** 1 Service d’Anesthésie-Réanimation du CHU Hédi Chaker de Sfax, Tunisie

**Keywords:** Bloc axillaire, anesthésie générale, hépatite A, kyste hydatique, Axillary block, general anesthesia, hepatitis A, hydatid cyst

## Abstract

Le bloc axillaire chez l'enfant est une technique facile et recommandée chez l'enfant. Sa réalisation chez un enfant présentant une hépatite A aigue n'est pas démunie de risques surtout lorsqu'il est associé à une sédation au propofol avec du remifentanil. De même, la présence d'un kyste hydatique unique laisse la possibilité de l'anesthésie générale avec ventilation uni pulmonaire.

## Abstract (en)

Axillary block is an easy and recommended technique in children. Its use in children with acute hepatitis A is not risk free especially when associated with sedation using remifentanil and propofol. Similarly, the presence of a single hydatid cyst allows general anesthesia with mono-pulmonary ventilation.

## Réaction

Je vous envoie cette lettre afin d'ouvrir le débat sur l'anesthésie locorégionale chez l'enfant et en particulier lorsque l'anesthésie générale est à risque suite à une publication précédente dans votre journal intitulée “Quand le bloc axillaire reste la seule alternative chez un enfant de 5 ans” par Mohamed Said Nakhli et al. [1]. Ce cas, présentant une hépatite A aigue avec un taux de prothrombine bas, a été considéré comme une contre indication à l'anesthésie générale alors que il existe des revues de la littérature qui ont montré que le risque de réduction du débit sanguin hépatique est de 30 à 50% quelque soit la technique anesthésique utilisée (générale ou locorégionale) [2]. Je rappelle que la préparation du matériel nécessaire pour une anesthésie générale fait partie des règles de sécurité lors d'une anesthésie locorégionalevu le risque de conversion [3]. Il me parait aussi que c'est un peu contradictoire de dire que le bloc axillaire reste la seule alternative anesthésique alors qu' une sédation au propofol-Remifentanil a été associée exposant au risque d'apnée et au recours à la ventilation mécanique [4]. En plus, le recours à un bloc axillaire est très fréquent en pédiatrie et c'est un bloc facile à réaliser et sa réalisation ne présente aucune originalité [5]. Cependant, il existe des publications à propos de blocs inhabituels chez l'enfant comme le bloc interscalénique [6] ou infra-claviculaire [7]. En outre, il me parait plus raisonnable d'utiliser la ropivacaïne 0,5% au lieu de la lidocaïne 1% [8]. Par ailleurs, le kyste hydatique du poumon de notre patient a été considéré comme à haut risque de fissuration proscrivant la ventilation mécanique alors qu'il s'agit d'un seul kyste, non compliqué, touchant un seul poumon laissant la possibilité de ventilation uni pulmonaire [9]. Je souhaitesavoir les dimensions de ce kyste ainsi que les critères de gravité exposant à la fissuration et je souhaite avoir une réponse sur votre stratégie de gestion de ce patient s'il a fait une apnée sous propofol-Remifentanil? Il me parait donc que le recours à l'anesthésie générale est inévitable dans ce cas et que le bloc axillaire n'est plus la seule alternative.

## Conclusion

Le bloc axillaire chez l'enfant est une technique largement utilisée en pédiatrie et permet une réduction de la consommation de morphiniques et une amélioration de l'analgésie [3]. Son utilisation chez un malade présentant des risques pour une anesthésie générale est recommandée mais n'est jamais la seule alternative et ne peut en aucun cas éliminer la possibilité d'une anesthésie générale.

## Conflits d’intérêts

Les auteurs ne déclarent aucun conflit d'intérêts.
